# Symptoms Relevant to Surveillance for Ovarian Cancer

**DOI:** 10.3390/diagnostics7010018

**Published:** 2017-03-20

**Authors:** Robert M. Ore, Lauren Baldwin, Dylan Woolum, Erika Elliott, Christiaan Wijers, Chieh-Yu Chen, Rachel W. Miller, Christopher P. DeSimone, Frederick R. Ueland, Richard J. Kryscio, John R. van Nagell, Edward J. Pavlik

**Affiliations:** 1 Division of Gynecologic Oncology, Department of Obstetrics and Gynecology, University of Kentucky Chandler Medical Center-Markey Cancer Center, Lexington, KY 40536-0293, USA; robert.ore@uky.edu (R.M.O.); labald1@uky.edu (L.B.); dylan.woolum@uky.edu (D.W.); erikatay28@gmail.com (E.E.); christiaan.d.wijers@vanderbilt.edu (C.W.); chieh-yu.chen@uky.edu (C.-Y.C.); raware00@email.uky.edu (R.W.M.); cpdesi00@uky.edu (C.P.D.); fuela0@email.uky.edu (F.R.U.); jrvann2@email.uky.edu (J.R.v.N.); 2 Department of Statistics, University of Kentucky Chandler Medical Center-Markey Cancer Center, Lexington, KY 40536-0293, USA; richard.kryscio@uky.edu

**Keywords:** symptoms, questionnaire, certainty/uncertainty

## Abstract

To examine how frequently and confidently healthy women report symptoms during surveillance for ovarian cancer. A symptoms questionnaire was administered to 24,526 women over multiple visits accounting for 70,734 reports. A query of reported confidence was included as a confidence score (CS). Chi square, McNemars test, ANOVA and multivariate analyses were performed. 17,623 women completed the symptoms questionnaire more than one time and >9500 women completed it more than one four times for >43,000 serially completed questionnaires. Reporting ovarian cancer symptoms was ~245 higher than ovarian cancer incidence. The positive predictive value (0.073%) for identifying ovarian cancer based on symptoms alone would predict one malignancy for 1368 cases taken to surgery due to reported symptoms. Confidence on the first questionnaire (83.3%) decreased to 74% when more than five questionnaires were completed. Age-related decreases in confidence were significant (*p* < 0.0001). Women reporting at least one symptom expressed more confidence (41,984/52,379 = 80.2%) than women reporting no symptoms (11,882/18,355 = 64.7%), *p* < 0.0001. Confidence was unrelated to history of hormone replacement therapy or abnormal ultrasound findings (*p* = 0.30 and 0.89). The frequency of symptoms relevant to ovarian cancer was much higher than the occurrence of ovarian cancer. Approximately 80.1% of women expressed confidence in what they reported.

## 1. Introduction

Intake forms are commonly used in clinical care and are often presented to women undergoing well-woman exams and routine gynecologic care. Guidelines exist for British general practitioners [1] as well as for American generalists [2] for collecting and evaluating information on symptoms related to ovarian cancer (OvCA). Women who report certain symptoms are candidates for testing with Ca125, pelvic ultrasound and/or referral to a gynecologic oncologist. Symptoms indicative of ovarian cancer have been included in information collected through the Patient Reported Outcomes Measurement Information System (PROMIS [3,4]) developed by NIH in the United States and integrated with electronic medical records in the ambulatory care setting [5]. Discrepancy has been described between clinician and patient symptoms reporting with many cancer-related symptoms going unrecognized [6]. The dynamics of communication between the physician and patient can be complex and lead to this discrepancy in symptoms discovery with the doctor assuming that the patient will initiate a revealing conversation while the patient expects the doctor to inquire about possible symptoms. Differences in symptoms reporting even exist between paper and electronic reporting [7].

The present report is unique in that it examines factors influencing personal confidence inherent to symptoms reporting by focusing on a large cohort of women without cancer. This report focuses on intake information specific to symptoms of ovarian cancer for deciding the possibility of malignancy. We have employed a questionnaire containing a constellation of symptoms (both related and not related to ovarian cancer) that was reported on by Goff [8]. While data challenging the power of this symptoms index to identify early-stage ovarian cancer has been reported [9,10], symptoms information cannot be ignored, otherwise delays in diagnosis can occur [11]. We have added a self-administered evaluation of reporting confidence to the Goff symptoms questionnaire in order to assess the degree to which women are confident in their responses and have analyzed serially completed questionnaires to determine how time and repeated exposure to symptoms reporting affect confidence. Contemplation of patient-reported confidence is paralleled by the judiciary system where a great deal of emphasis is placed on witness confidence in determining the credibility of testimony [12]. Our report is noteworthy because it identifies changing patient confidence in information that they report on questionnaires which should make physicians more sensitive to the reliability of patient responses.

## 2. Materials and Methods

Women enrolled in the ongoing ultrasound-based University of Kentucky Ovarian Cancer Screening Program [13,14,15] from 1987 to July 2013 consisted of both women in the general population and those of high risk based on confirmation of a primary or secondary relative diagnosed with ovarian cancer (*n* = 41,529). Approval was received from the University of Kentucky Institutional Review Board (IRB number 88-0021-9F6, renewed 11 August 2016). Women were recruited by physician recommendation, media announcements, and word of mouth. Women needed to be competent and understand the terms of the informed consent presented in English, or they were excluded from screening.

Participants in this screening program are characterized as health conscious (>90% medical checkups, >85% annual mammography), well educated (>50% college, ~3% not high school graduates), married (75%) and medically insured (95%) [16].

In October of 2008, participants began completing a modified symptoms questionnaire printed in English which was originally developed by Goff et al. [8]. In total, 24,526 women completed the questionnaire and 17,623 women completed the questionnaire more than once on subsequent screens, for a total of 70,734 evaluated questionnaires. The questionnaire was in the exact form as published by Goff, [8] but was modified to include the confidence of the responder as reported [9]. This modification added the question: “How confidently did you answer these questions?” The possible responses were: “*no confidence*” = 0, “*minimally sure*” = 1, “*more than minimally sure*” = 2, “*pretty sure*” = 3, “*sure*” = 4 and “*absolutely sure*” = 5. The screening sonographer queried each participant about their understanding of each symptom and was responsible for the participant providing answers to all data fields prior to screening. Sonographers gave explanations about the symptoms on the questionnaires as a clarification process prior to screening. Effort was made to model general clinical practice by presenting clarifications as necessary at every participant encounter with the questionnaire. The setting for this study was most similar to women presenting for well-woman exams or routine gynecological checkups. Each questionnaire was completed prior to screening ultrasonography. Over the course of the study 12 different sonographers were involved, each of which received individual training related to questionnaire administration.

Study eligibility, exclusions, instrumentation, protocol, criteria for designating an abnormality, data collection and storage were as previously reported [14,17,18,19]. In brief, criteria for eligibility were: (1) women aged ≥50 years and (2) women aged 25–49 years with a documented family history of OvCA in at least one primary or secondary relative.

Participants provided their medical history, surgical history, menstrual history/menopausal status, hormonal use, and family history of cancer. Women with a known ovarian tumor or a personal history of OvCA were excluded. Ultrasound findings were designated as abnormal if ovarian volume exceeded 20 cm^3^ for pre-menopausal women or 10 cm^3^ for post-menopausal women, and if cysts (with septations, solid areas, or papillary projections) as well as echogenic solid structures were observed. An abnormal screening result referred exclusively to the ultrasound result *per se* and not to biomarkers or genetic testing results. Less than 100 women were observed to have free fluid on their ultrasound exam and free fluid generally resolved on their subsequent exam(s) so that free fluid was not treated as an informative predictor.

Following an abnormal ultrasonographic result, repeat screens were scheduled at intervals ranging from six weeks to six months and the symptoms questionnaire was re-administered at each screening. In the present study, the majority of screens were administered annually. The mean interval between questionnaires was 1.15 years ± 0.01 (SEM), median = 1.03 years, min = 0.02 years/max = 4.9 years, 75th percentile = 1.13 years, 90th percentile = 1.49 years, 95th percentile = 1.95 years. Criteria for *Goff symptoms* related to ovarian cancer were a symptom presenting for >12 days per month with an onset <12 months for having pelvic or abdominal pain, being unable to eat normally, feeling full quickly, feeling abdominal bloating or increased abdominal size. Symptoms *unrelated to ovarian cancer* included on the Goff questionnaire (*non-Goff symptoms*) used in the present study were: back pain, indigestion, nausea, vomiting, weight loss, urinary urgency, frequent urination, constipation, diarrhea, menstrual irregularities, bleeding after menopause, pain during intercourse, fatigue, leg swelling, difficulty breathing.

Confidence of respondents on the symptoms questionnaire was examined in terms of age, menopausal status, body mass index (BMI), hormone replacement therapy (HRT) usage, reporting *no* vs. *any* symptoms, number of Goff symptoms reported, number of non-Goff symptoms reported, number of any symptoms reported and receipt of an abnormal ultrasound screening result. Subjects with missing information listed above were excluded.

### Statistical Methods

All information was entered by the sonographer performing the ultrasound into a Medlog database (Medlog Systems, Crystal Bay, NV, USA) using encodings for symptoms, severity, frequency & duration to minimize error on an electronic template organized identically to the printed questionnaire. Random audits of the data and corrections yielded estimates of accuracy greater than 98%. Significance was determined at the *p* ≤ 0.05 level in order to robustly identify differences. Proportions were compared using chi-square statistics. In longitudinal analysis, McNemars test for correlated proportions in the marginals was used.

*Multivariate analysis*: Two binary variables were created from the symptoms confidence scores (CS): (1) *no confidence* defined as a confidence score of 0 versus all other (higher) scores and (2) little confidence defined as a score of 0 or 1 versus all other (higher) scores. Each was tabulated against the assessment number. It was decided to abbreviate the assessment number as 1, 2, 3, 4, or 5 plus assessments on the basis of the sample size for each value and due to the fact that the percentage of respondents with no or little confidence did not vary much beyond the fifth time the confidence score was recorded. Similar cross tabulations were done for other potential explanatory variables including BMI (recorded as less than 25, 25–29.99, or 30 plus); presence of HRT (yes or no); number of reported Goff symptoms complying with frequency (>12 days/month) and duration (<12 months) recorded as 0, 1, or 2 plus; abnormal screen (yes or no); menopausal status (premenopausal, postmenopausal, or peri-menopausal); and the number of other symptoms (non-Goff symptoms, recorded as 0, 1–10, and ≥11). Age at the assessment was not recoded.

To compare the percentage of “no” or “little” confidence scores among assessments, a generalized linear mixed model was constructed based on a logit link function. Confidence was rated on a six-point Likert (ordinal) scale. The model was fitted using a generalized estimating equation (GEE) procedure to account for repeated assessments on the same subject (working correlation matrix estimated using a compound symmetry assumption). This was done for both a reduced model with only assessment number as a predictor variable and then for a full model with all variables outlined above used as predictor variables. Because the results for the assessment variable were similar for each model, we report only the results for the full model. Statistical significance was determined at the 0.05 level. The GEE models were fitted using PROC GENMOD in PC-SAS, Version 9.3 (SAS Institute, Cary, NC, USA).

## 3. Results

The demographic characteristics of the group studied are presented in Table 1. None of these women had a diagnosis of ovarian malignancy during the study period or during 40 months of follow-up. Only a small fraction (7.1%) experienced an abnormal ultrasound exam during the study period during which they completed symptoms questionnaires. A total of 24,526 women completed 70,734 symptoms questionnaires (Table 2). The vast majority of participants (prevalence = 88.8%) at some time reported one or more of the constellation of symptoms with only 11.2% never reporting any symptom, shown in Table 2. About a third of reported symptoms (31.9%) occurred on the first questionnaire, while 68.1% had no symptoms on the first reporting. Only 11.5% did not report any symptoms after reporting symptoms on the first report, while about twice as many (20.7%) continued to report symptoms, shown in Table 2. A majority (67.8%) reported symptoms after not having symptoms on the first reporting, accounting for a 60.2% incidence, shown in Table 2. More than 9500 women completed the symptoms questionnaires four or more times, accounting for more than 43,000 symptoms questionnaires completed four or more times (Table 3). Examination of reported confidence on the symptoms questionnaires was made with confidence considered as both a confidence score >0 and >1.

Confidence (CS > 0) was highest on the first questionnaire completed (83.3% of all respondents) and decreased to 74% when five or more questionnaires were completed (Table 4). Complete lack of confidence (CS = 0) in symptoms reporting was observed in 21.1% of all responses and increased (from 16.7% to 26%) as a function of questionnaires completed (Table 4, CS = 0 line), showing decreasing confidence despite increasing experience with the symptoms questionnaire.

### 3.1. General Factors Associated with Expressions of Confidence in Symptoms Reporting

With increased age, a statistically significant decrease in confidence in symptoms reporting was observed (Table 5), with the fall-off appearing after age 60 so that the ratio of confident to non-confident women over 75 years (2.0) was half that of women under 40 (4.0), shown in Table 5.

For women under age 40, 80.1% (859/1073) expressed confidence in their response and this decreased to 76.1% for all women over 40 (53,007/69,661), shown in Table 5. Confidence decreased to 75.9% (50,658/66,750) for women over 50, to 73.4% (33,084/45,082) for women over 60 and to 68.9% (11,565/16,778) for women over 70 (*p* < 0.0001). Expressed confidence for postmenopausal women was 75.7% (49,100/64,831), mirroring confidence for women over 50 years of age.

The fraction of underweight (BMI ≤ 18.5) and normal weight (BMI = 18.5–24.9) women who expressed confidence in their reporting (21,263/27,932 = 76.1%) was not significantly different from overweight (BMI = 25–29.9) and obese (BMI ≥ 30) responders (32,603/42,802 = 76.2%). The fraction of women that received an abnormal screening result and expressed confidence in their reporting only differed by 1% from the fraction of women that had a normal screening result, while for only Goff symptoms the difference was 6% and not statistically significant.

Significantly more women reporting at least one symptom expressed confidence in their responses (41,984/52,379 = 80.2%) than women who reported no symptoms (11,882/18,355 = 64.7%), *p* < 0.0001. Women that reported at least one Goff symptom relevant to ovarian cancer expressed confidence with the same frequency (1597/1931 = 82.7%) as women that did not report any Goff symptoms (9895/11,871 = 83.4%). There were more women that expressed confidence who reported at least one of the symptoms (those not relevant to ovarian cancer) (37,163/45,992 = 80.8%) than women who did not report any symptoms (16,703/24,742 = 67.5%), *p* < 0.0001. Thus, participants that were the least certain about what they reported were those women who did not report having symptoms.

### 3.2. Longitudinal Analysis of Confidence Stability

**Figure 1:**
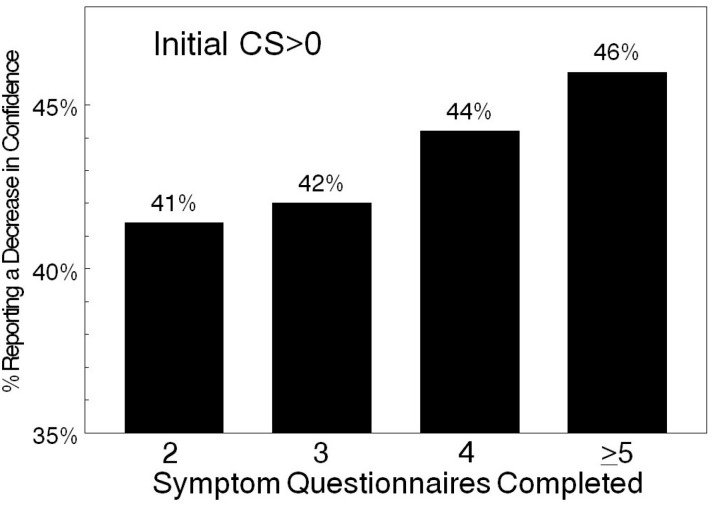
Confidence reported as a function of the number of symptoms questionnaires completed. Decreased confidence reported by women who originally reported confidence (CS > 0).

Efforts were directed at determining if confidence scores changed as individuals completed more symptoms evaluations. Analysis focused on 17,623 individuals who completed two or more symptoms questionnaires. Results were based on individuals initially reporting some confidence (CS > 0) and tracked on the basis of the number of symptoms questionnaires that were completed. The change between the first and last confidence score was determined for each individual as increasing, decreasing or unchanged. The fraction of women that demonstrated a decrease in confidence expanded as additional questionnaires were completed (Figure 1). Confidence remained unchanged in approximately one-third of the cases (35.1%–37.4%, Table 6). Confidence scores increased in ~20% of women that initially reported some confidence (CS > 0: 18.4%–22.6%, Table 6). Decreases in confidence occurred in just under 50% of the individuals that initially reported some confidence (CS > 0: 41.4%–46%, Table 6). There was a statistically significant difference in the response distribution between individuals completing the questionnaire two to three times vs. those taking the questionnaire five or more times (*p* < 0.005), shown in Table 6. Examining paired longitudinal differences using the McNemars test showed a significant difference (*p* < 0.0001) for completing three, four, or five or more evaluations compared to two evaluations (Table 6). Thus, longitudinal analysis indicated a trending decrease of confidence scores (Table 6) in almost half of the women completing the symptoms questionnaires.

### 3.3. Multivariate Analysis

Relating the binary outcome (confidence scale) to the number of symptoms questionnaires completed was based on the frequencies reported in column 2 of Table 3 and not on arbitrarily varying the cut point to achieve significant results. The percentage of respondents expressing no confidence increased significantly from 16.7% after the first assessment (*p* < 0.0001 when each of the no confidence levels for assessments two, three, four, or five plus were compared to the first assessment). It then leveled off during assessments two, three, or four (23.0%, 22.7%, and 23.4%, respectively) which were not statistically different from each other. However, by assessment five or later, those expressing no confidence increased to 26.0% which is significant when compared to assessments two, three, or four (*p* < 0.001 in all cases). All other variables examined were significant in the multivariate model except for use of hormone replacement therapy (*p* = 0.44), and normal vs. abnormal screening exams (*p* = 0.09). Thus, although the number of women with abnormal findings is small, so it should be expected to have little effect in this study, it does not test as a confounder. Specifically, the percentage of patients expressing no confidence increased with age (*p* < 0.001). The percentage was stable through age 60 and then increased steadily from 18.8% to 32.8% by age 85; decreased for morbidly obese patients (19.9% compared to normal BMI 21.2%, (*p* < 0.03); declined with the number of other symptoms reported (symptoms unrelated to ovarian cancer) from 31.2% (score 0) to 18.5% (scores 1 through 10) to 6.4% (score 11); decreased with the number of reported Goff symptoms complying with frequency (>12 days/month) and duration (<12 months) from 21.3% at score 0 to 15.1% at score 1 to 12.1% for scores ≥2; and increased in postmenopausal women when compared to premenopausal women (21.3% versus 19.3%, *p* < 0.0001). Similar results were obtained for the endpoint *little confidence* (results not shown).

### 3.4. Symptoms Reported Relevant to Ovarian Cancer

Overall, 59.9% (42,404/70,734) of the symptoms questionnaires reported one or more of the five symptoms related to ovarian cancer, but only 3.9% (2756/70,734) met the frequency and duration criteria and did so with a significantly different distribution (Table 7. *p* < 0.0001). The overall incidence of symptoms was: abdominal bloating > pelvic pain > increased abdominal size > feeling full quickly > unable to eat normally (Table 7). In these women that were not diagnosed with an ovarian malignancy during the study period or during 40 months of follow-up, the incidence of any of the five symptoms relevant to ovarian cancer was high, but frequency and duration information significantly reduced this number. Symptom severity was significantly lower in women that did not meet the Goff-positive frequency and duration criteria (*p* < 0.001, Table 7), but did not differ with regards to reported confidence (CS = 0 vs. CS > 0). Most women (68.4%, Table 8) reported only one symptom that met the Goff criteria of frequency and duration, while 23.3% reported two and ~8% reported three or more of these symptoms (Table 8). Moreover, the incidence of symptoms was not different with respect to reported confidence (CS = 0 vs. CS > 0). Nevertheless, the 2.7% Goff-positive occurrence (Table 8: 1931/70,734) was nearly ~245 times higher than the ovarian cancer incidence for this population (11.2/100,000), [20]. Unlike one-time reports that have previously considered symptoms related to ovarian cancer, the present report is a longitudinal study of multiple reports collected over time. Consequently, a woman may be positive for the Goff ovarian cancer symptoms in the context of always meeting or sometimes meeting the frequency and duration criteria. There are also women in the present data set who, after being positive for the Goff ovarian cancer symptoms, subsequently no longer report these symptoms. Against this background, to address these considerations, we identify two groups: (A) women that at any time have reported any Goff ovarian cancer symptoms and (B) women that at any time satisfied the frequency and duration criteria for any Goff ovarian cancer symptoms. Approximately one-third of the women surveyed (7983/24,526) qualified for inclusion in Group A, while ~7% of women qualified for inclusion in Group B (1708/24,526). Our estimates mirror a recent report from the United Kingdom on ovarian cancer symptoms reported in the general population [21]. In relating these findings to the positive predictive value (PPV) which depends on prevalence (PPV = True Positives/(True Positives + False Positives)), the work presented here would yield a symptoms-estimated PPV of 0.073% or one malignancy for 1368 cases that would be taken to surgery using the sample reported on here (24,526 women filling out 70,734 questionnaires reporting 52,467 symptoms for 21,789 women) and screen-detected ovarian cancers reported previously [9]. This symptoms-estimated PPV is smaller than that reported by Rossing from a much smaller study size (*n* = 1905) [10] that would not have approached prevalence as closely as the results described here. However, despite the occurrence of symptoms being vastly higher than the incidence of ovarian cancer, ignoring symptoms is very likely to result in women being diagnosed with advanced-stage disease [11].

## 4. Discussion

This is the first work to examine symptoms related to ovarian cancer in a very large sample and to consider the confidence that women, all with an eventual non-surgical outcome, have in the responses they entered on a symptoms questionnaire that they completed prior to their ultrasound exam. A significant finding of the work presented here is that a large majority of women (80.1%) were confident in their reporting. Confidence was lowest (64.7%) in women who did not report any symptoms. Decreasing confidence despite increasing experience with the questionnaire was demonstrated by the finding that the fraction lacking confidence increased as a function of the number of times that the symptoms questionnaire was completed. Importantly, confidence scores in individuals followed longitudinally showed a decreasing trend in almost 50% of women. There was a significant age-related decrease in confidence, and women that did not report any symptoms were significantly less confident than women who reported at least one symptom. Importantly, confidence decreased as more symptoms were reported, including both ovarian cancer–related Goff symptoms complying with frequency (>12 days/month) and duration (<12 months), as well as other symptoms unrelated to ovarian cancer. Thus, reporting of an increased number of symptoms did not coincide with greater confidence in the results reported. Analyses of symptom severity indicated that severity was higher in women that met the Goff-positive frequency and duration criteria than in women that did not, suggesting that transient or long-standing symptoms may be of lower intensity. It is noteworthy that symptoms reporting was done prior to receiving an ultrasound exam with the result that there was no statistically significant difference in confidence between women receiving a normal vs. abnormal sonographic result.

These findings indicate that while *uncertainty in symptoms reporting* occurs to a much lesser extent than certainty, every individual’s report must be carefully assessed and not unconditionally accepted. It may even be appropriate to consider serial evaluation of symptoms in order for physicians to understand the extent to which complaints continue to persist or resolve. The symptoms questionnaire utilized here includes reporting of frequency and duration in addition to the actual symptoms. Consequently, uncertainty about frequency and duration may be contributing to how an individual’s response reflects confidence in what they report on the questionnaire. Memory certainly plays a role in recalling when symptoms began and how often they have occurred, and this may become more challenging as a person gets older. Thus, age-related effects on memory may be most relevant to certainty about the frequency and duration of symptoms and, with multiple co-morbidities that accumulate over time, can make it difficult to identify a “new” symptom per se or to pinpoint its onset. It is also possible that as a person gets older, they become accepting of many of the symptoms considered here occurring sporadically or episodically and as such are reluctant to declare them a symptom of anything other than age.

An impact on the healthcare delivery system arises when symptoms related to ovarian cancer are reported by women that do not have an ovarian malignancy and can result in inappropriate clinical decisions that could lead to unnecessary surgery. Some data exist supporting symptoms-based surveillance with even early cancers producing symptoms detectable by questionnaire [22]. Symptoms reporting is currently important for the identification of patients needing imaging and closer examination. Just as a lack of witness confidence in legal testimony raises questions about credibility, physicians should be sensitive to the same possibility being relevant to over-diagnosis and over-treatment if a patient may be uncertain about what they report. In addition, certainty about symptoms should not be mistaken to be related to the presence of pathology. Physicians should be made aware that confidence will decrease with age and that reporting multiple symptoms does not imply patient confidence or credibility in the report. Thus, physicians should deliberate through patient information in order to make appropriate assignments of diagnostic tests and follow-up.

The strengths of this study include the large number of patients participating, and the large number of patients completing questionnaires on more than one occasion. In addition, trained sonographers assisted participants in collecting their medical history by answering questions about the context of the questionnaires that participants were filling out. The present report focuses on the level of confidence women have in reporting symptoms as a statistical estimation and not hypothesis testing. It investigates factors that might alter this level and while this involves hypothesis testing, the large sample size assures adequate statistical power to identify some factors that do affect the reported confidence level.

The inherent weakness of a study of this nature is its subjective nature. One person’s symptom may be something that someone else has become accustomed to. Subjectivity also occurred in the confidence scale; however, its gradation allowed different dichotomization points to be examined to delineate certainty from uncertainty. It is also possible that a lack of confidence associated with reporting an increased number of symptoms reflects a lack of confidence in only part of the symptoms reported on the questionnaire but not in others. This possibility was not examined in the design that was utilized because addressing this would add the burden of 63 individual confidence assessments (i.e., confidence assessments for 21 symptoms, amplified by confidence queries on severity, frequency and duration: 21 × 3 = 63). Understanding the context of the questionnaire certainly has an influence on confidence. The questionnaire used here included reporting of severity, frequency and duration in addition to the symptoms per se. Consequently, uncertainty about severity, frequency and duration may contribute to how an individual response reflects confidence.

Directions for future study might include an assessment of whether the levels of confidence reported here are chiefly related to completing a printed questionnaire and how they also extend to interviews with healthcare professionals. The discrepancy between clinician and patient symptoms ratings is greatest for more subjective symptoms [23]. To this end, it must be realized that clinician symptom ratings are lower than patient-reported ratings [24,25]. Consequently, care must be taken about assuming the superiority of information on symptoms gathered by clinicians and about the inferiority of patient-reported symptoms. Likewise, the results here indicate that uncertainty can exist in patient-reported symptoms.

## 5. Clinical Implications

Although the balance between patient confidence and uncertainty very heavily favors confidence, the level of *uncertainty in symptoms reporting* described here should be kept in mind when extracting symptoms information from patients. This principle may affect the extent to which symptoms information is relied upon or should be probed during the clinical evaluation process. The addition of psychosocial tools to evaluate the contributions of stress, anxiety and depression need to be explored to help the clinician extract the pertinent information from patient symptoms reporting so that those most at risk for malignancy can be identified.

## Figures and Tables

**Table 1:** Demographic characteristics of the study group at first symptom evaluation.

Variable	All, *n* = 24,526 Women
Age	61.7, 61 (24–99)
Parity	2.3, 2 (1–19)
Weight (pounds)	162.4, 156 (76–420)
Height (inches)	64.3, 64 (47–78)
BMI	27.6, 26.6 (12.6–80.5)
Family history of:	
Ovarian cancer	5566 (22.7)
Breast cancer	10,935 (44.6)
Colon cancer	6595 (26.9)
Personal history of:	
Breast cancer	2278 (9.3)
Colon cancer	202 (0.8)
No history of hormone replacement therapy	21,206 (86.5)
History of hormone replacement therapy	3315 (13.5)
Nulliparous	3500 (14.3)
Premenopausal	1597 (6.5)
Perimenopausal	444 (1.8)
Post menopausal	22,840 (93.1)
Abnormal exam history	1742 (7.1)
Any symptoms	18,610 (75.9)
Goff symptoms	845 (3.4)
Other symptoms	16,433 (67.0)

Data are mean, median (range) or *n* (%). BMI: body mass index.

**Table 2:** Frequency and occurrence of symptoms.

Duration Period of Data Collection Studied 15 April 2008–25 June 2013	
Women screened	24,526 (100%)
Symptoms questionnaires administered	70,734 (100%)
Questionnaires reporting symptoms	52,467 (64.3%)
Women reporting symptoms	21,789 women (88.8%) on 52,467 questionnaires
Women never reporting symptoms	2737 (11.2%)
Women reporting symptoms on first symptoms questionnaire	6956 (31.9% of women reporting symptoms)
Women reporting symptoms with no symptoms on first symptoms questionnaire	14,833 (68.1% of women reporting symptoms)
Women reporting symptoms on first symptoms questionnaire AND subsequently no symptoms reported	2503 (38.2% of women reporting symptoms on 1st questionnaire; 11.5% of all women reporting symptoms)
Women reporting symptoms on first symptoms questionnaire AND subsequently symptoms reported	4515 (68.9% of women reporting symptoms on 1st questionnaire; 20.1% of all women reporting symptoms)
Women reporting NO symptoms on first symptoms questionnaire AND subsequently symptoms	14,771 (99.6% of women with no symptoms on 1st questionnaire; 67.8% of women reporting symptoms)

**Table 3:** Frequency of symptom questionnaire completion.

Number of Symptoms Questionnaires Completed	Women Completing Questionnaire (*n*)	Total Questionnaires Completed
1	6903	6903
2	4423	8846
3	3696	11,088
4	4530	18,120
5	4168	20,840
6	714	4284
7	84	588
8	7	56
9	1	9
Total	24,526	70,734

**Table 4:** Confidence as a function of the number of symptoms questionnaires completed.

Confidence	Questionnair Completed	Nunber Completed	Nunber Completed	Nunber Completed	Nunber Completed	Total Completed
Confidence Score (CS)	1st	2nd	3rd	4th	5 or more	All times
0	4103 (16.7)	4055 (23)	2992 (22.7)	2226 (23.4)	1529 (26)	14,905 (21.1)
1	714 (2.9)	443 (2.5)	391 (3)	250 (2.6)	165 (2.8)	1963 (2.8)
2	506 (2.1)	411 (2.3)	349 (2.6)	226 (2.4)	172 (2.9)	1664 (2.4)
3	4090 (16.7)	1984 (11.3)	1353 (10.3)	989 (10.4)	593 (10.1)	9009 (12.7)
4	4280 (17.5)	3477 (19.7)	2774 (21)	2127 (22.4)	1289 (21.9)	13,947 (19.7)
5	10,833 (44.2)	7252 (41.2)	5341 (40.5)	3686 (38.8)	2134 (36.3)	29,246 (41.3)
Responses	24,526 (100)	17,622 (100)	13,200 (100)	9504 (100)	5882 (100)	70,734 (100)
Women completing	1	2	3	4	≥5	Questionnaires
*n*	6903	4423	3696	4530	4974	24526
Comparisons 1 vs. 2,3,4 or >4	*p* < 0.0001					
2 vs 3, 4		NS *p* > 0.5				
2, 3, 4 vs. >4		*p* < 0.0001				

Response scores were: “no confidence” = 0, “minimally sure” = 1, “more than minimally sure” = 2, “pretty sure” = 3, “sure” = 4 and “absolutely sure” = 5. Analysis for difference included both 0 vs. all other scores and 0 + 1 vs. all other score in both 2 × 2, 2 × 6, 2 × 5 contingency tables. NS: not statistically significant.

**Table 5:** Confidence as a function of age.

Age, Years		Confidence *n* (%)		Y/N Ratio
	Women	N = No	Y = Yes	
25–40	1073 (1.5)	214 (19.9)	859 (80.1)	4.0
41–50	2911 (4.1)	562 (19.3)	2349 (80.7)	4.2
51–60	21,668 (30.6)	4094 (18.9)	17,574 (81.8)	4.3
61–74	35,900 (50.8)	8972 (25)	26,928 (75)	3.0
≥75	9182 (13)	3026 (33)	6156 (67)	2.0
Total	70,734 (100)	16,868	53,866	

**Table 6:** Longitudinal stability as a function of the number of symptoms questionnaires completed (CS > 0).

Questionnaires Completed	Change	*n*	%	Comparison	Significance
2	a. Increased	827	22.6%	2 vs. 3, 4	NS
2	b. Unchanged	1318	36.0%	2 vs. ≥5	*p* < 0.005
2	c. Decreased	1518	41.4%		
2	Sub-total	3663	100.0%		
3	a. Increased	688	22.3%	3 vs. 4	NS
3	b. Unchanged	1101	35.7%	3 vs. ≥5	*p* < 0.005
3	c. Decreased	1297	42.0%		
3	Sub-total	3086	100.0%		
4	a. Increased	708	18.4%	4 vs. ≥5	NS
4	b. Unchanged	1439	37.4%		
4	c. Decreased	1702	44.2%		
4	Sub-total	3849	100.0%		
≥5	a. Increased	793	18.8%	4 vs. ≥5	NS
≥5	b. Unchanged	1478	35.1%	3 vs. ≥5	*p* < 0.005
≥5	c. Decreased	1936	46.0%	2 vs. ≥5	*p* < 0.005
≥5	Sub-total	4207	100.0%		

Significance in the table is based on chi square 3 × 2 contingency table analyses. *p* < 0.0001 using McNemars test for correlated proportions in the marginals of a 2 × 2 contingency table for initial confidence >0 where decreased paired confidence = ”Yes”. Comparisons were for two to five or more evaluations. Odds ratio changed from 1.18 (two vs. three evaluations) to 1.496 (two vs. five or more evaluations). *p* < 0.0001 using McNemars test for initial confidence = 0 where increased confidence = “Yes”.

**Table 7:** Occurrence of symptoms related to ovarian cancer.

**Symptom**	**Goff-Negative Occurrence Freq < 12 per Month and Duration > 12 Months, *n* (%)**	**CS = 0**	**Severity**	**CS > 0**	**Severity**
Pelvic Pain	10,859 (25.6)	1702 (24.3)	2.1 ± 0.03	9157 (25.9)	2.1 ± 0.01
Unable to eat normally	2584 (6.1)	459 (6.6)	2.2 ± 0.06	2125 (6)	2.2 ± 0.03
Feeling full quickly	5566 (13.1)	960 (13.7)	2.2 ± 0.04	4606 (13)	2.1 ± 0.02
Abdominal bloating	14,934 (35.2)	2477 (35.4)	2.2 ± 0.02	12,457 (35.2)	2.2 ± 0.01
Increased abdominal size	8461 (20)	1396 (20)	2.3 ± 0.03	7065 (20)	2.3 ± 0.02
Total	42404 (100)	6994 (100)		35,410 (100)	
**Symptom**	**Goff-Positive Occurrence Freq > 12 per Month and Duration < 12 Months, *n* (%)**	**CS = 0**	**Severity**	**CS > 0**	**Severity**
Pelvic Pain	588 (21.3)	86 (22.6)	3.1± 0.13	502 (21.1)	3.04 ± 0.05
Unable to eat normally	244 (8.9)	36 (9.5)	3.1 ± 0.21	208 (8.8)	3.5 ± 0.09
Feeling full quickly	446 (16.2)	62 (16.3)	3.3 ± 0.15	384 (16.2)	3.2 ± 0.06
Abdominal bloating	832 (30.2)	115 (30.2)	3.5 ± 0.1	717 (30.2)	3.4 ± 0.04
Increased abdominal size	646 (23.4)	82 (21.5)	3.4 ± 0.13	564 (23.8)	3.12 ± 0.05
Total	2756 (100)	381 (100)		2375 (100)	

Severity was reported using the scale: 1 = minimal to 5 = severe (mean ± SEM). Severity Goff-negative vs. Goff-positive: *p* < 0.001.

**Table 8:** Occurrence of multiple symptoms.

Number of Symptoms	Goff-Positive Occurrence Freq > 12 per Month and Duration < 12 Months, *n* (%)	CS = 0	CS > 0
1	1321 (68.4)	200 (73)	1121 (67.7)
2	450 (23.3)	49 (17.9)	401 (24.2)
3	115 (6)	18 (6.6)	97 (5.9)
4	35 (1.8)	6 (2.2)	29 (1.8)
5	10 (0.5)	1 (0.4)	9 (0.5)
Total	1931 (100)	274 (100)	1657 (100)

CS = 0 vs. CS > 0: *p* = 0.23.
